# Supplementary material for: MADS-Box Family Genes in Lagerstroemia indica and Their Involvement in Flower Development
Source: Plants (Basel). 2024 Mar 1;13(5):709. doi: 10.3390/plants13050709 (PMC10934564; doi:10.3390/plants13050709)
Supplement: Supplementary file 1 [file Figure S1.pdf › Figure S1.pdf]

(a)

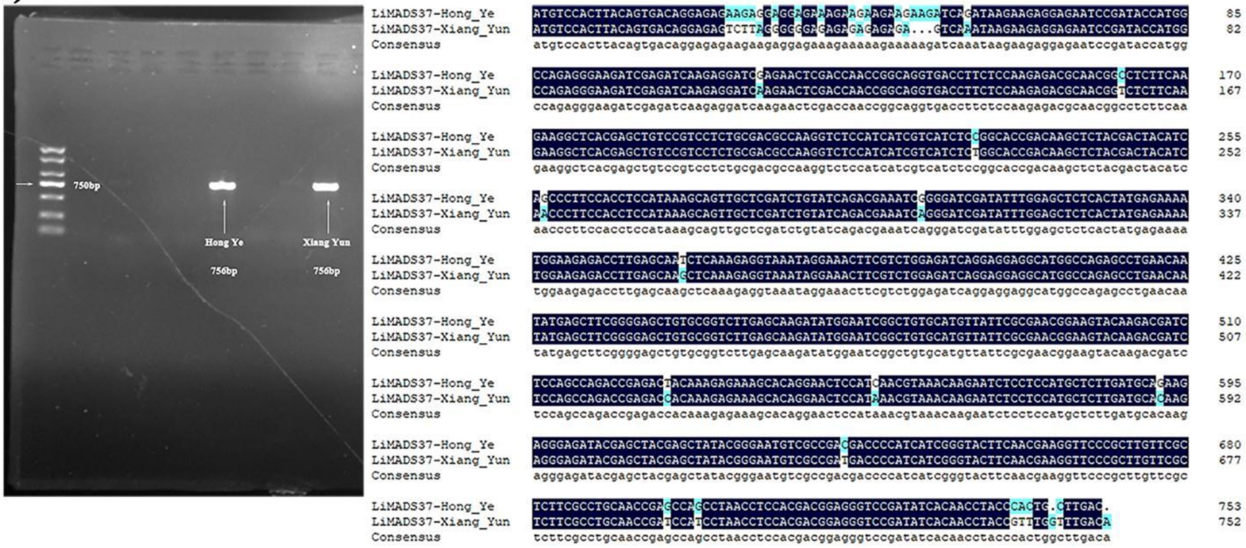

(b)

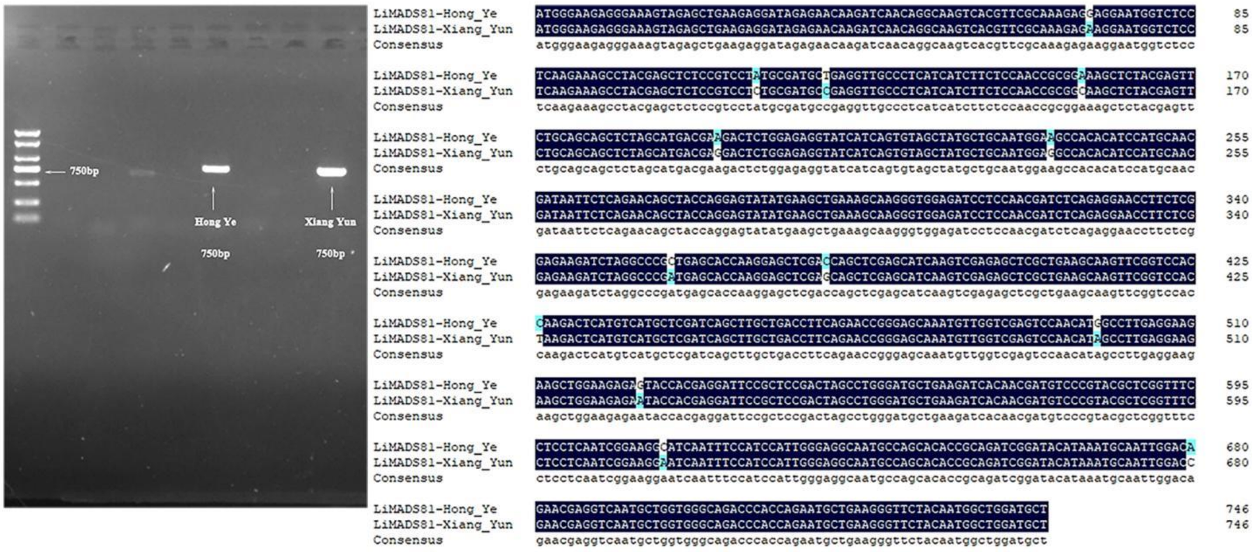

Figure S1. Sequence differences between Xiang Yun and Hong Ye genes. (a) LiMADS37 gel electrophoresis plot with sequence comparison; (b) LiMADS81 gel electrophoresis plot with sequence comparison.
